# Individual, unit and vocal clan level identity cues in sperm whale codas

**DOI:** 10.1098/rsos.150372

**Published:** 2016-01-20

**Authors:** Shane Gero, Hal Whitehead, Luke Rendell

**Affiliations:** 1 Zoophysiology, Department of Bioscience, Aarhus University, Aarhus, Jutland, Denmark; 2 Department of Biology, Dalhousie University, Halifax, Canada B3H 4J1; 3 Centre for Social Learning and Cognitive Evolution, and Sea Mammal Research Unit, School of Biology, University of St Andrews, St Andrews KY16 9TH, UK

**Keywords:** social complexity hypothesis, conformism, individuality, communication, social structure, cetaceans

## Abstract

The ‘social complexity hypothesis’ suggests that complex social structure is a driver of diversity in animal communication systems. Sperm whales have a hierarchically structured society in which the largest affiliative structures, the vocal clans, are marked on ocean-basin scales by culturally transmitted dialects of acoustic signals known as ‘codas’. We examined variation in coda repertoires among both individual whales and social units—the basic element of sperm whale society—using data from nine Caribbean social units across six years. Codas were assigned to individuals using photo-identification and acoustic size measurement, and we calculated similarity between repertoires using both continuous and categorical methods. We identified 21 coda types. Two of those (‘1+1+3’ and ‘5R_1_’) made up 65% of the codas recorded, were shared across all units and have dominated repertoires in this population for at least 30 years. Individuals appear to differ in the way they produce ‘5R_1_’ but not ‘1+1+3’ coda. Units use distinct 4-click coda types which contribute to making unit repertoires distinctive. Our results support the social complexity hypothesis in a marine species as different patterns of variation between coda types suggest divergent functions, perhaps representing selection for identity signals at several levels of social structure.

## 1. Introduction

Complex social structure may be an evolutionary driver of communication systems according to the ‘social complexity hypothesis’ [1]. Species which live in large and highly organized societies are expected to exhibit more complex communicative signals [1–6]. While communication complexity reaches a peak in primates with human language, the correlation between social complexity and greater variation in communicative signals has been demonstrated in several different taxa, including bats [7], non-human primates [8,9], mustelids [10] and birds [11]. These patterns could result from an individual’s need to navigate a wide range of social interactions by identifying themselves to conspecifics as members of broader social groups, in order to interact effectively and efficiently. Should this be the case, social complexity would drive selection for social signals which function in mediating social recognition.

Cetaceans are an important taxon for testing the social complexity hypothesis as they have cognitive capacities [12], communication systems [4,13] and social structures [14] that rival their terrestrial counterparts in complexity, while providing a dramatic contrast in ecology. The cetaceans’ marine habitat varies greatly over long spatial and temporal scales and less over smaller ones in comparison to terrestrial systems [15], resulting in an environment in which social learning may be especially adaptive over typical marine mammal lifespans [16]. Accordingly, the communicative and cognitive characteristics of cetaceans, along with their highly variable ecological and social environments, seem to have favoured the cultural transmission of behaviour patterns; and consequently much group-level behavioural variation [17,18].

One might expect that the variability in vocal signals in cetaceans would be the greatest where recognition of social identity is most important and that different signals might face different selective pressures for differing functions. Here, we examine evidence of this in the sperm whale (*Physeter macrocephalus*, Linnaeus 1758), a cetacean which has a particularly interesting culturally transmitted communication system and a multilevel social structure including what may be the largest cooperative groups of vertebrates outside of humans [19,20]. While mature males tend to live relatively solitary lives, there are several hierarchical tiers of female social structure. One or more matrilines form social ‘units’ whose female membership is stable across decades [21–23]. Units range widely, covering distances up to 2000 km [24,25]. ‘Groups’ are formed when units associate for periods ranging from hours to days. Sperm whales produce social calls termed ‘codas’ that are stereotyped patterns of three or more broadband clicks [26]. Codas appear to function in social communication in that they are often overlapped and exchanged between individuals in duet-like sequences [27], and they are produced at high rates during periods of social behaviour at the surface [28] but not during foraging at depth [29]. Distinct coda dialects delineate vocal clans—collections of units that share a similar dialect which can number in the thousands of individuals. In the Pacific Ocean, where multiple clans live sympatrically, their distinct culturally transmitted dialects appear to socially segregate their society, since units only associate with each other if they share a dialect [20,30]. In the Atlantic Ocean, however, the picture is different. Coda repertoires appear to vary geographically, and in general, only one repertoire is heard in any given area [19,31].

The observed diversity of coda types appears excessive however if they serve only to identify social structure at the clan level—Rendell & Whitehead [20] identified over 70 types but only five vocal clans. The variation in coda production at the level of the individual and unit therefore demands closer examination in order to understand the evolution of an individual’s repertoire of communication signals. This study investigated the coda production of individuals from nine social units from the eastern Caribbean using an unparalleled dataset across a 6-year study (2005–2010). We quantified variation between units and individuals at two scales: (i) by their repertoire of codas (i.e. variation in the presence, absence and pattern of usage of various coda types) or (ii) by structural variation within given coda types (i.e. variation in the acoustic characteristics of specific coda types which are shared among units or individuals).

## 2. Material and methods

### 2.1 Field methods

Social units of female and immature sperm whales were observed in a 2000 km^2^ area along the entire western coast of the island of Dominica (N15.30 W61.40). Social units were delineated as in previous work based on association data collected over the 6 years of the study [23]. Research was conducted in the winters of 2005 through 2010 for a total of 2549 h with whales across 324 days of effort. (See the electronic supplementary material, table S1 and [23] for a full description of field methodology.)

Acoustic recordings were made of the diving whales for two purposes: (i) to record the first echolocation clicks of singleton diving whales (i.e. a cluster containing only one whale) for measuring that individual’s echolocation click inter-pulse interval, and therefore size [32,33] (see below) and (ii) to record coda output for clusters of all sizes while initiating dives. Codas were also recorded when the whales were socializing in groups at the surface.

### 2.2 Analyses

#### 2.2.1 Assigning codas to individuals

Sperm whale clicks typically contain multiple pulses with decaying amplitude which result from reverberations within the nasal complex of the whales [34,35]. By the nature of the sound’s path, the inter-pulse interval (IPI) has a direct relationship with the size of the spermaceti organ [33,36–43].

Here, we use IPI measurements to identify vocalizing individuals. Three steps were required to identify and assign vocalizations to individuals: (i) recordings of the first few minutes of echolocation clicks of photoidentified singletons were used to define the echolocation click IPI for that individual, (ii) analysis of the coda recordings to determine the coda click IPI for each coda using the same methods as for the singleton echolocation clicks, and (iii) using the library of echolocation click IPIs (see the electronic supplementary material, pp. 2–4), assignation of a coda to an individual whale when its modal echolocation click IPI (derived from the recordings taken when the individuals were alone) was within 0.05 ms of the modal coda click IPI of a whale which was present at the time of recording and at least 0.1 ms different from the modal coda click IPI of every other whale present at the time of the recording. This analysis used methods developed by Schulz *et al.* [33] with some minor modifications (see full details in the electronic supplementary material).

#### 2.2.2 Measuring and testing of similarity between repertoires

To define the temporal structure of the codas recorded, we measured absolute inter-click intervals (ICIs, the time between the onset of one click and the onset of the next click; see the electronic supplementary material for methodological details and analytical pathway). To quantify similarity between repertoires of individuals or units, we used two different measures: one categorical and one continuous.

For the categorical measure of similarity, two codas were given a similarity of 1 if they were assigned to the same type and were given a similarity of 0 if they were assigned to different types. We assigned codas of similar click length to a categorical type using a hierarchical clustering algorithm called OPTICS [44]. OPTICS produces a linear ordering such that the points closest in multivariate space become neighbours in the ordered list. Distances between neighbours are calculated and the algorithm uses the contrast parameter *ξ* to define a cut off limit for drops in distance (or ‘reachability’) to define distinct clusters (details in [44]). We used a *ξ* value of 0.04 for all coda lengths (which defines a 4% drop in point density as the criterion for defining a new cluster) as this best defined the clusters visually evident in plots of the first two components of a principal components analysis run on the same data. OPTICS was run on absolute ICI measures. OPTICS allows for codas that are outliers or located in sparse areas between dense clusters to be labelled as noise, rather than being forced into clusters. This creates a scenario in which classification of click patterns is highly conservative (all codas included in a cluster—i.e. a coda type—are very similar to each other), and ambiguous codas were labelled as ‘noise’. All codas which were not classified into clusters and labelled as noise by OPTICS were omitted from the categorical analysis but retained in the classification-free continuous measure of similarity described below. We used the OPTICSxi module in the ELKI framework (http://elki.dbs.ifi.lmu.de/) [45] to run these analyses.

Coda types were given names based on the mean temporal click pattern for all codas included in that cluster, following previous nomenclature [20,33,46]. For example, a ‘5R’ coda is one in which five clicks are regularly spaced, while a ‘1+1+3’ coda sounds like ‘click-[PAUSE]-click-[PAUSE]-click-click-click’ with longer gaps between the first two clicks followed by three clicks in quick succession. In addition, we calculated the Shannon index (*H*) [47] to estimate coda type diversity of individual repertoires, following previous work on call diversity [48,49].

For the classification-free, continuous measure, we calculated the multivariate similarity of two codas with the same number of clicks using either the Euclidean or infinity-norm distance between the ICI vectors of those codas. The summation of these distances to produce a measure of similarity between two coda repertoires was calculated in the same way as previous studies (complete details in [50] and the equation used is outlined in the electronic supplementary material). Similarities were calculated using custom-written routines in Matlab v. 7.12 (The Mathworks, Inc., MA, USA).

Matrix correlations and Mantel tests with 10 000 permutations [51,52] were used to test repertoire variation between similarity matrices of individuals within units, and between units, where a unit’s repertoire was simply all the codas produced by individuals belonging to that unit (details in the electronic supplementary material). In addition, to compare temporal changes in unit and individual repertoires, we calculated the mean similarity values and their standard errors between recordings of a focal unit or individual within and between years. All matrix correlations and Mantel tests were carried out using SOCPROG v. 2.5 [53] in Matlab v. 7.12 (The Mathworks, Inc., MA, USA).

We then used similarity matrices between repertoires to construct average-linkage clustering dendrograms and we tested their robustness using 1000 bootstrap replicates. The cophenetic correlation coefficient was also calculated to indicate how well the dendrogram represented the data [54].

To determine whether particular coda types could be used to discriminate between units or individuals, we undertook several discriminant function analyses (DFAs) on subsets of the data: only codas with four clicks, only codas with five clicks, only the ‘1+1+3’ codas (which is the most common coda type recorded in the study), all the ‘5R’ patterned codas (Antunes *et al*. [55] previously suggested individual variation in the 5R coda type), and on the two most common types defined by OPTICS which have five clicks and a regular rhythm (‘5R_1_’ and ‘5R_2_’; we chose to exclude ‘5R_3_’ due to the small sample size and it being largely produced by only one unit). Linear DFA was conducted on absolute ICI data, with all codas classified as ‘noise’ by OPTICS removed. Given that correct classification may be biased due to imbalances in the sample sizes between the groups being discriminated (i.e. the expectation under random assignment for *n* groups is not simply 1/*n* if the sample sizes are not equal), we further tested the classification by comparing the observed correct classification rate against those from 1000 randomized datasets. To do this, we randomly re-assigned codas to either individual or unit, depending on the analysis level, while keeping the number of codas made by each individual or unit constant. We then recorded the mean correct classification from the 1000 random datasets. Full details of implementation are found in the electronic supplementary material.

## 3. Results

**Figure 1.**
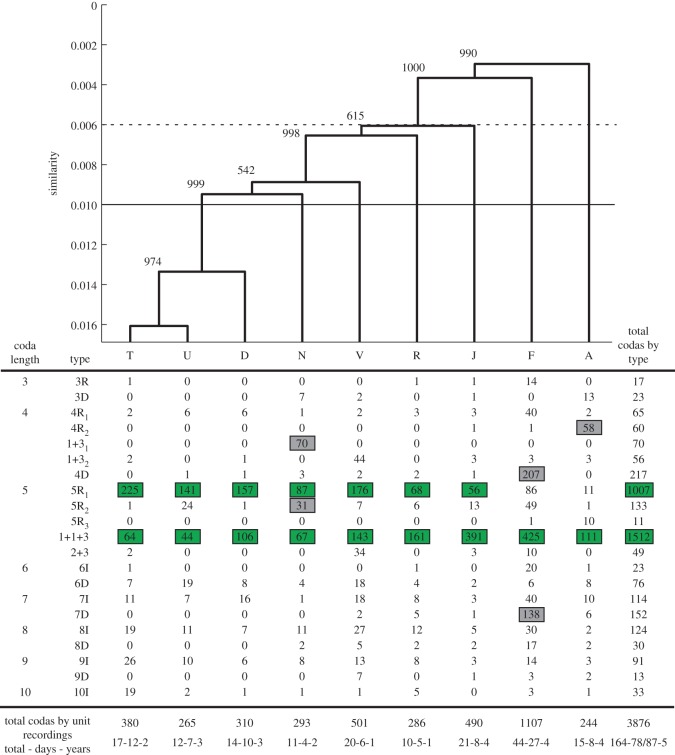
Coda repertoires of units of sperm whales recorded off Dominica compared using Euclidean distances of absolute ICIs with a basal similarity of 0.001 (top) and OPTICS categorical classification into types (bottom). Numbers next to branches of the dendrogram indicate the number of the 1000 bootstrap replicates in which that branch was reproduced. This is a good representation as the dendrogram has a cophenetic correlation coefficient of 0.9707. Horizontal rules indicate the mean between (dotted) and within (solid) unit similarities. Letters denote units. Numbers in the table indicate the frequency with which each individual coda type was produced by each unit. Shaded numbers indicate predominant coda types which made up at least 10% of the unit’s coda production. Numbers shaded in green indicate predominant types shared across units and shaded in grey where unit specific. For the nomenclature: ‘R’ indicates a coda with regular ICIs, ‘+’ indicates a longer gaps between clicks, ‘D’ indicates decreasing ICIs throughout the coda, ‘I’ indicates increasing ICIs throughout the coda, and the sequential numbering of the same name (e.g. 5R_1_, 5R_2_, 5R_3_) indicates coda types with the same rhythm but of increasing duration. Numbers below each column are the total number of codas recorded from each unit, as well as the total number of recordings, recording days, and years per unit. On 8 days recordings were made of different units; as a result, that day was counted once as a recording day for each unit in the unit totals. There is therefore a difference in the two overall totals for days. The slash in the overall total divides Unique Calendar Days/Unit Days.

We made more than 10 recordings over more than 4 days for each of nine social units: A, D, F, J, N, R, T, U and V, with seven of these recorded in multiple years (figure 1). Units varied in size (mean =8 individuals, range =4–12), but all contained at least one calf (details of composition in [23]). We attributed 4116 codas to the nine units across 164 recordings in five different years. A total of 243 (5.9%) codas were excluded as noise by OPTICS and the remaining 3876 codas were categorized into 21 types (see the electronic supplementary material, figure S2, for a plot showing rhythms of all types). Two particular types made up 65.0% of the codas recorded (‘1+1+3’—39.0% and ‘5R_1_’—26.0%). The next most common type, ‘4D’, made up only 5.6% of all codas. Overall, 4- and 5-click codas made up 82% of all codas. For three of the units (F, J and U), we were able to reliably assign each coda to the individual producing it, giving a dataset of 1502 codas assigned to 18 individuals. Discovery curves suggest that repertoires at both the unit and individual level were well characterized (electronic supplementary material, figure S3). Our results were robust to varying the distance measure used to quantify multivariate similarity in codas. Below, we present the data using the Euclidean distance measure only, as the infinity-norm distances produced similar patterns (see the electronic supplementary material, tables S6 and S7 for infinity-norm results)

### 3.1 Differences between units

Mantel test across all units tested the null hypothesis that repertoire similarity between recordings of the same unit on different days (within unit) was the same as that between recordings of different units on different days (between units). Our results confirmed that units differ consistently in the repertoire of codas which they use (multivariate similarity: matrix correlation =0.17,*p*<0.001; categorical similarity: matrix correlation =0.15, *p*<0.001; table 1). However, all units have two or more coda types which each make up more than 10% of their coda production (shaded boxes in figure 1). All units produced large numbers of the ‘1+1+3’ coda and all but two units (A and F) produced many of the ‘5R_1_’ coda as well. Four of the nine units (A, F, N and V) each produce a distinctive 4-click coda type. Unit N is the only unit to use type ‘5R_2_’ (a coda with the same rhythm as but markedly slower tempo than 5R_1_) as more than 10% of its repertoire, although it is produced by all other units. The production of these additional types by certain units creates much of the structure in the average-linkage cluster dendrogram from the unit-level analysis (figure 1). The overall cascading pattern of the dendrogram suggests little distinction between the repertoires of all units and the mixed levels of bootstrap support among T, D, V, U and N concur with this interpretation. Furthermore, units did not significantly alter their repertoires over time, at least across the 6 year duration of this study, as the mean within-year similarity is systematically not greater than that between years (electronic supplementary material, table S2).

**Table 1.** Mean repertoire similarities within and between units and individuals. Mantel test across all units has a null hypothesis that repertoire similarity between recordings of the same unit on different days (within unit) is the same as that between recordings of different units on different days (between units). Mantel tests among individuals within the three units have a null hypothesis that repertoire similarity between recordings of the same individual on different days (within individual) is the same as that between recordings of different individuals on different days (between individuals). Multivariate similarity using absolute ICIs, Euclidean distances and a basal similarity of 0.001. Categorical similarity using OPTICS classification.

	multivariate similarity				categorical similarity			
units	within	between	matrix correlation	*p*-value	within	between	matrix correlation	*p*-value
across units	0.010	0.006	0.17	<0.001	0.292	0.200	0.15	<0.001
individuals in F	0.023	0.003	0.57	<0.001	0.549	0.173	0.40	<0.001
individuals in J	0.016	0.007	0.36	<0.001	0.739	0.435	0.25	<0.01
individuals in U	0.034	0.010	0.64	<0.001	0.546	0.214	0.50	<0.001

**Figure 2.**
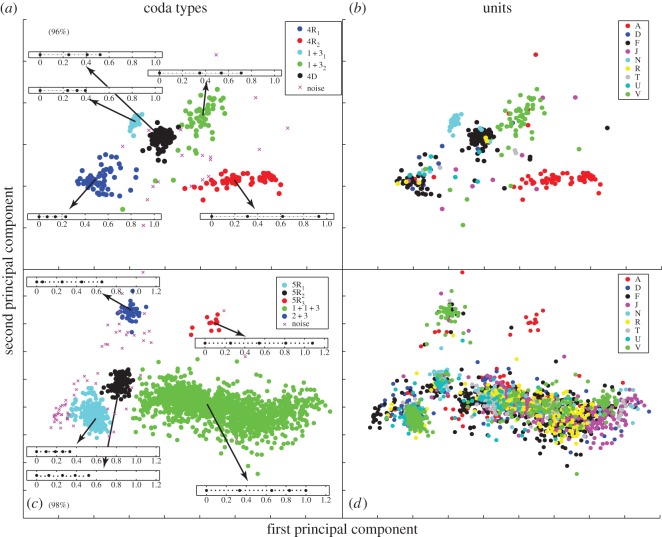
First two principal components for all (*a*,*b*) 4-click codas and (*c*,*d*) 5-click codas. Panels (*a*,*c*) are coloured by coda type with exemplar rhythms inset with duration given in seconds.Panels (*b*,*d*) are coloured by social unit. Percentage in parentheses denotes the variance explained by the first two principal components.

**Figure 3.**
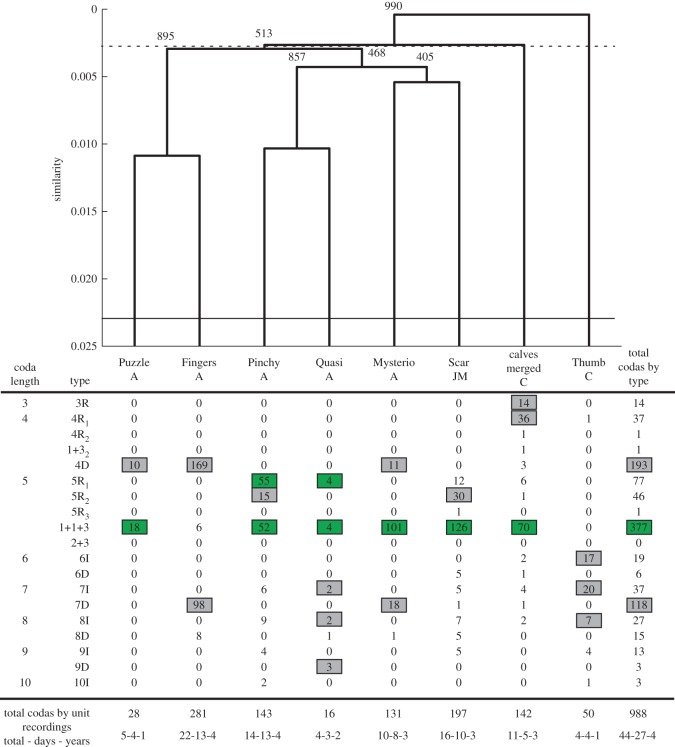
Coda repertoires of individuals in Unit F compared using Euclidean distances and absolute inter-click intervals with a basal similarity of 0.001 (top) and OPTICS classification into types (bottom). Numbers next to branches of the dendrogram indicate the number of the 1000 bootstrap replicates in which that branch was reproduced. A cophenetic correlation coefficient of 0.8626 suggests this is overall a good representation of the data, but low bootstrap support for some branches suggests fine-level clustering may not be significant. Horizontal rules indicate the meanbetween (dotted) and within (solid) individual similarities. Letters denote age class (A, adult; JM, juvenile male; C, calf). Numbers in the table indicate the frequency with which each coda type was produced by each individual. Shaded numbers indicate predominant coda types which made up at least 10% of the individual’s coda production. Numbers shaded in green indicate common predominant types identified in the unit-level analysis (figure 1). All notations are as in figure 1.

Each of the four units that produce large numbers of 4-click codas use a different 4-click coda type (Unit N: ‘1+3_1_’, Unit V: ‘1+3_2_’ Unit F: ‘4D’ and Unit A: ‘4RL’); which results in a discriminant function with a correct classification of 88% for 4-click codas to units (figure 2*a* and table 2, standardized discriminant coefficients and variance explained can be found in the electronic supplementary material, table S10). The increased production of these coda types by the respective units was consistent across years, so cannot be the result of context-specific sampling on single occasions. It is less clear whether the distinct 4-click coda types are in each case produced by a single member of the unit with a distinctive individual repertoire, or whether each is a part of a shared repertoire. Individual-specific coda repertoires were only available for one of these units, Unit F. Three of the adult females in the unit used the 4D coda type in different years and calves from this unit also produced it, suggesting it is shared, but it is also the case that one of the three females (‘Fingers’) produced it more frequently and consistently than the other two in every year (figure 3).

**Table 2.** Significance tests, DFA correct classification on observed data, and correct classification based on randomized data; where *n* is the number of units or individuals. All combinations tested can be found in the electronic supplementary material, tables S8 and S9.

coda dataset	Wilks *Λ*	approx. F	d.f.	*p*-value	randomized correct (%)	DFA correct classification (%)
4-click coda types; *n*=9 units	0.045	106.42	24	<0.001	54	88
1+1+3 codas; *n*=14 individuals	0.495	9.347	52	<0.001	19	33
1+1+3 within unit F; *n*=6 individuals	0.507	11.22	20	<0.001	37	57
1+1+3 within unit J; *n*=4 individuals	0.733	7.54	12	<0.001	38	51
1+1+3 within unit U; *n*=3 individuals	0.827	0.920	8	0.505	63	58
All 5R1; *n*=12 individuals	0.223	11.59	44	<0.001	21	46
5R1 within unit F; *n*=3 individuals	0.526	6.156	8	<0.001	73	83
5R1 within Unit J; *n*=2 individuals	0.648	6.925	4	<0.001	54	73
5R1 within unit U; *n*=3 individuals	0.373	19.138	8	<0.001	51	78
5R1 within unit V; *n*=3 individuals	0.140	11.731	8	<0.001	38	79

By contrast, discriminant functions were not able to reliably classify codas above chance levels to units across all 5-click codas or using either of the two most common coda types (‘1+1+3’ and ‘5R_1_’), with correct classifications of only 29%, 34% and 30%, respectively, which are similar to those produced by random assignment (table 2). Standardized coefficients of all DFAs can be found in the electronic supplementary material (figures S10, S11 and S12).

### 3.2 Patterns across individuals

Mantel tests among individuals within the three units tested the null hypothesis that repertoire similarity between recordings of the same individual on different days (within individual) is the same as that between recordings of different individuals on different days (between individuals). The results demonstrate that coda repertoires differed between individual members of all three units (table 1 and figure 3 for Unit F, and in the electronic supplementary material, figures S4 and S5 for units J and U, respectively). The general pattern emerging from all three units is that individuals universally shared both the ‘1+1+3’ and at least one of the 5R-type codas, but they differed in their usage of coda types and also in the way they produced certain types. The DFA within the 5R_1_ coda type revealed that when discriminating individual members within units, we could correctly classify 73%, 78% and 83% of these codas to the individual that produced them for Units J, U and F, respectively (table 2). While we were not able to construct full repertoires for individuals in Unit V, a DFA had similar success when discriminating a small subset of 5R_1_ codas by three individuals from this unit (table 2). When all individuals across units are pooled, the DFA is still significant, but correct classification is reduced to 46%. The significance of the Wilks’ test for the DFA but low proportion of correctly classified codas at this level suggests that while there is individual variation in the 5R_1_ coda type, reliably identifying individuals is only possible within units.

By contrast, we were not reliably able to discriminate individuals across units using all of the 5-click coda types, all of the 1+1+3 codas, or when we included the 5R_2_ codas with the 5R_1_. These DFAs only resulted in correct classifications of 29%, 33% and 46%, respectively. Furthermore, unlike with the 5R_1_ codas, we were not able to discriminate individuals within units using the 1+1+3 codas (table 2; highest correct classification of 58%). Standardized coefficients of all DFAs can be found in the electronic supplementary material, tables S10–S12.

The mean similarities within and between years of an individual’s repertoire (electronic supplementary material, table S2) did not differ significantly indicating that individuals do not change their vocal repertoire between years, at least over the 6 year duration of this study (2005–2010). Furthermore, adult females kept their vocal repertoires consistent through changes in parental role including after births of new dependent calves and, in one case, the loss of a calf (see the electronic supplementary material, tables S3–S5 for examples).

Juveniles and calves appear to use more coda types than adults (adults: mean=4.9 types, range =2–9, mean Shannon index, *H*=1.28, range =0.10–2.45; juveniles/calves: mean=8.6 types, range=2–18, mean Shannon index, *H*=1.89, range =0.91–2.42). In particular, all of the calves in the three units used the 4R1 while only three of the 12 adults produced this type. Given the difficulty in assigning codas to specific calves when several are present, the vocal production of the two living calves in Unit F was merged. Interestingly, their merged vocal repertoire differed from that of ‘Thumb’, the only calf in Unit F in 2005 who was a male and estimated to be only three months old [56] when recordings of him were made before his death between the 2005 and 2006 field seasons (figure 3). The other living calves (both also male) were older, ranging in age from 1 to 5 between 2008 and 2010 when their codas were recorded, and had repertoires more similar to that of the adults in their natal social unit.

## 4. Discussion

This study shows that coda production acoustically marks various levels of sperm whale social structure in different ways. Taken in combination with their well-studied, multi-levelled social structure [19] and previous work at the level of the clan [20], the variation in levels of specificity (number of individuals discriminated by particular calls or cues within call types) and multiplicity (the number of different call types) found here provides support for the ‘social complexity hypothesis’ among sperm whales and broadens the taxonomic range over which the hypothesis has been supported to include a marine species.

While we have found statistical differences in vocal repertoires among individual sperm whales and the social units to which they belong, overall coda production was dominated by the ‘1+1+3’ and ‘5R_1_’ coda types. Repertoire similarity between units in the Caribbean (0.006, s.e. =0.0001) is far higher than for units which are members of the same vocal clan in the Pacific (0.002, s.e. <0.0001) (Pacific values re-calculated using Euclidean distance, as in this study, from data reported in [20]). It appears, therefore, that all of the units in the eastern Caribbean belong to one vocal clan, as defined in the Pacific [20]. This is consistent with the picture of geographical variation of repertoires in the Atlantic [19,31], in that we have only found a single repertoire in the Caribbean and no evidence of sympatric dialects.

What variation there is between units appears to be primarily the result of four of the nine units (A, F, N and V) regularly using characteristic 4-click coda types in addition to the 1+1+3 and the 5R_1_. We noted that two of those four units (N and V) used variations on the 1+3 rhythm, which has obvious qualitative similarity to the 1+1+3 coda type. It is difficult to interpret these observations because while those units produced large numbers of these 4-click codas, every unit had at least one 4-click coda in their sampled repertoire, but they were mostly produced in very low numbers. There was nothing obviously different about the units that did and did not use 4-click codas heavily, such as size or the presence of calves, for example. It is difficult therefore to distinguish without further data whether such patterns reflect a genuine variation in unit-specific coda production; although, within the data available, 4-click coda use was consistent across multiple recordings and years.

The most striking finding is the differing patterns of variation within different coda types. We have shown that all the units, and the individuals within them produced the ‘1+1+3’ coda in a stereotyped way, such that we cannot detect systematic variation between units or individuals. Previous studies have documented the production of the 1+1+3 coda type in the eastern Caribbean over the last 30 years. Recordings made in the 1980s [57] and 1990s [58] were also dominated by these same coda types. While 30 years is within the lifespan of many of the adult females in this study (longevity more than 70 years [59]) and long-term re-sightings have identified one individual in Unit D off Dominica from as far back as 1984 [23], the younger animals (born during this 6-year study) also produce this coda in the same way. The 1+1+3 coda’s stability over this timeline and ubiquity across a population divided into disparate social units provides a rare example of a cultural transmission maintaining high levels of conformity of a behaviour.

By contrast, we also found a significant level of individual variation in the production of the ‘5R_1_’ coda type. This is consistent with an emerging picture of individual differences in how the ‘5R’ is produced [55,60]. These differing patterns between coda types would suggest that variation in codas serves different functions. We propose that different classes of coda may vocally differentiate three levels of social structure. While ‘5R_1_’ codas may be used for individual discrimination within units, the characteristic 4-click codas may serve to identify unit membership in specific contexts, and the ‘1+1+3’ coda could likewise function as a marker of vocal clan. This type of hierarchical recognition is common in bird song, in which the general form of the song identifies the species while variations within it can identify individuals [61,62]. Interestingly, the ‘5R’ type appears to be a ubiquitous coda, being reported in several studies from the eastern tropical Pacific [20,63], Japan [64] and across the Atlantic [31], while the ‘1+1+3’ has only ever been reported from the eastern Caribbean. Furthermore, the 1+1+3 codas appear to cluster distinctly when comparing all 5-click codas from across the Atlantic Ocean (see Type #51 in fig. 5.3 of [31], p. 81). These patterns further support this functional interpretation. While previous work has demonstrated the broad patterns expected under the social complexity hypothesis [7–11], here we demonstrate that the expected functional diversity needed to mediate the specific complexities of multiple social tiers in sperm whales society is addressed by the according variability or stereotypy in the social identity cues found in their communication system.

For any vocal recognition system to function, it must have three key properties [65]: (1) signals which vary and/or are produced with sufficient stereotypy to provide identity information, (2) receivers which can distinguish between these signals, and (3) receivers which respond differently to signallers based on their identity and interaction history. Previous work has shown that sperm whales structure their associations at the individual [66], unit [67] and vocal clan levels [20] across both short [66] and long time scales (at least decades [67]) suggesting that they do respond differently to their conspecifics’ identities (evidence of (3) above). This study has demonstrated the presence of identity cues in sperm whale codas and furthermore that the required variation in specificity (number of individuals classified by particular calls) and multiplicity (the number of different calls for a given level of structure) exists with which to discriminate among several tiers of their social structure (evidence of (1) above).

It is important here to distinguish between discrimination based on identity cues and identification based on identity signals. Identity cues are features of a trait that allow for the discrimination of identity by human researchers and/or the animals themselves; while identity signals are the product of selection favouring the ease of identification by conspecifics [68,69]. The existence of cues does not imply that selection has acted on those cues to produce unambiguous, recognizable identity signals. In this study, we were able to reliably discriminate between individuals within a given unit of sperm whales by identity cues in their ‘5R_1_’ coda. This does not necessarily establish that they are identity signals, as we lack two crucial pieces of knowledge. The first is whether receivers can distinguish the variability within and between coda types that we have identified and/or the extent to which the features of codas that we have measured here map onto the features that are salient for the whales themselves ((2) above). This issue is difficult to determine, but we argue that the temporal structure measured here is likely to be important, because it is the aspect of signal production that is most resistant to change due to variations in oceanic sound transmission, ambient pressure at the depth of production and directionality of frequency content. However, it is also possible that sperm whales themselves can extract other forms of information from the codas such as the signaller’s IPI, as well as adding that information to their pre-existing knowledge of the spatial and social relationships between nearby conspecifics at the time of production [70]. The second area of missing knowledge is the extent to which the sperm whales are in fact using these vocal identity cues to mediate their diverse social relationships. In order to gain a full understating of the function of sperm whale codas, further research is needed in order to understand the social and behavioural context in which particular coda types are exchanged and to undertake playback experiments such as those which confirmed bottlenose dolphin signature whistles as vocal labels [71–73].

Lastly, this study has also provided further insight into how individual sperm whale coda repertoires develop. When communication signals are acquired through social learning, individuals often have a period of increased diversity and low stereotypy, often referred to as ‘babbling’, before converging on the precise production of the adult repertoire [74–76]. Schulz *et al.* [33] suggested its occurrence among sperm whales based on recordings of a single calf. Our larger dataset supports this suggestion with a more general finding that juveniles and calves produce a higher diversity of coda types. Moreover, we show that it takes several years for young calves to consistently produce the typical repertoire of their natal unit. Furthermore, the coda types used by adults and those used by calves are subtly different which may suggest the gradual acquisition of adult coda types through some form of learning, as has been documented among songbirds and human babies [77].

## 5. Conclusion

Individual differences in vocalizations are ubiquitous among animals, but the degree of stereotypy we document in one socially learned coda type (1+1+3) among an entire population of sperm whales, and its stability across at least 30 years, is rare. This provides a remarkable example of cultural transmission maintaining high levels of conformity in behaviour across large numbers of individuals that are not continuously associated. The differences in patterns of variation between the two most common coda types suggest divergent functions, perhaps representing selection for identity labels at varying levels of social structure. While functional explanations for these hierarchical levels of variation are necessarily speculative, this study shows that various levels of sperm whale social structure can be distinguished through vocal variation. This work thereby broadens the taxonomic range over which the social complexity hypothesis has been supported by indicating that selection for advertising identity at different levels of a society may contribute to the evolution of a more complex vocal repertoire.

## Supplementary Material

Full multipage ESM in one file covering extended methods and supplementary results.

## Data accessibility

The ICI data used in this study is available from the Dryad Digital Repository: http://dx.doi.org/10.5061/dryad.ck4h0.
