# Supplementary material for: Individual, unit and vocal clan level identity cues in sperm whale codas
Source: R Soc Open Sci. 2016 Jan 20;3(1):150372. doi: 10.1098/rsos.150372 (PMC4736920; doi:10.1098/rsos.150372)
Supplement: Full multipage ESM in one file covering extended methods and supplementary results. [file rsos150372supp1.docx]

Supplementary Material for Gero *et al.* (XXXX). Individual, unit, and vocal clan level identity cues in sperm whale codas. RSOS

**Field Effort:**

Field work was undertaken for a total of 2549 hours with whales across 324 days of effort on one of three platforms: a dedicated 12m auxiliary sailing vessel, a dedicated 5m outboard skiff, or an 18m whale-watch vessel. Effort is broken down by year and platform in Table S1

Table S1: Effort across years

| **Year** | **Start Date** | **End Date** | **Days Effort** | **Platform** |
| --- | --- | --- | --- | --- |
| **2005** | January 14 | April 13 | 62 | Sailing only |
| **2006** | January 17 | February 11 | 21 | Whalewatch only |
| **2007** | January 28 | February 28 | 30 | Skiff and Whalewatch |
| **2008** | February 8 | May 8 | 75 | All |
| **2009** | January 11 | March 29 | 64 | Skiff and Whalewatch |
| **2010** | January 20 | April 18 | 72 | Sailing only |

**Recording Systems:**

Vocalizations were recorded using one of several recording setups: In 2005, we used a Fostex VF-160 multitrack recorder (44.1 kHz sampling rate) and a custom built towed hydrophone (Benthos AQ-4 elements, frequency response: 0.1-30kHz); no recordings were made during the short 2006 season; in the 2007 and 2009, we used a Zoom H4 portable field recorder (48 kHz sampling rate) and a Cetacean Research Technology C55 hydrophone (frequency response: 0.02-44kHz); during the 2008 and 2010 seasons, we used the custom-built towed hydrophone (Benthos AQ-4 elements, frequency response: 0.1-30kHz) and computer based recording system as a part of the International Fund for Animal Welfare’s (IFAW) LOGGER software package (48 kHz sampling rate). As we use only the temporal patterning of clicks in this analysis, variation in the frequency responses of the recording systems used is not important.

**Analytical Pathway**

In our multi-stage analysis, we used two measures to quantify the ICIs within codas, two methods of comparing similarity between repertoires, and two distance metrics to quantify multivariate similarity between pairs of codas. We took this approach in order to account for our decisions made during analysis and to demonstrate that our results were robust to analytical choices. In figure S1, we plot the flowchart of our analysis. In the main text, we present the results using the absolute ICIs, both the categorical and continuous similarity measures, and using the Euclidean distance metric for multivariate similarity between codas. In the ESM, we provide similar results using standardized ICIs and an infinity-norm distance metric (see tables S6 and S7).


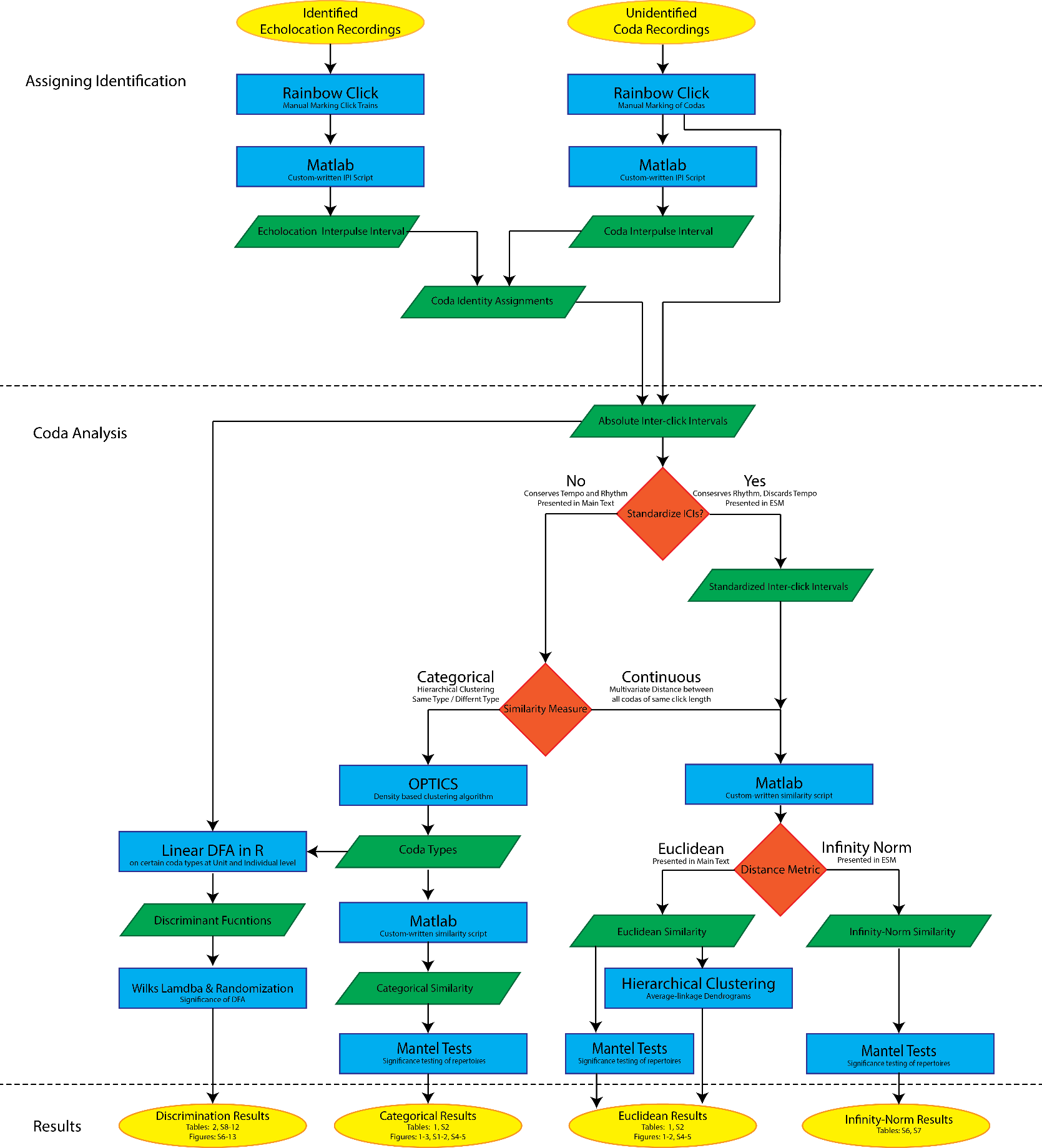


Figure S1 – Analytical pathway from raw data (upper yellow ovals) to results (lower yellow ovals) through both assigning codas to identified individuals and the coda repertoire and discrimination analysis. Blue rectangles are analytical procedures, green parallelograms are data input or output, red diamonds are parameter or metric decisions.

**Justification for the use of OPTICSxi over previously used k-means methods**

We selected the OPTICSxi algorithm over the *k*-means clustering used in previous studies [1–3] because analyses have shown that OPTICS’ density-based approach is both more successful at defining natural clusters in large test datasets and does so more accurately than *k*-means [4]. Part of the improved functionality of OPTICS is that it allows for points that are outliers or located in sparse areas between dense clusters to be labelled as noise, rather than being forced into defined clusters. This creates a scenario in which classification of click patterns is highly conservative (all codas included in a cluster – i.e. Type – are very similar to each other), and ambiguous codas were removed from the categorical analysis as ‘noise’.

### **Classification-free Similarity of Repertoires**

We calculated the similarity between coda repertoires, *A* and *B* (produced either by two units or two individuals depending on the level of analysis), in the same way as previous studies [1–3]:

where *S_AB_* is the similarity between repertoires *A* and *B* each with *n_A_* and *n_B_* codas, respectively; *l_i_* and *l_j_* are the number of clicks in coda *i* from repertoire *A* and the number of clicks in coda *j* in repertoire *B*; *b* is the basal similarity [here set to 0.001 seconds, which gives a very fine scale comparison of codas on the order of 1 ms, but the results are robust to variation in b, see 3,5] and *d_ij_* is either the Euclidean or infinity-norm distance between the ICI vectors of codas *i* and *j*. The equation implicitly assigns multivariate similarity between two codas containing different numbers of clicks to zero. Similarities were calculated using custom-written routines in *MATLAB* 7.12 (The Mathworks, Inc., MA, USA).

### **Assigning Codas to Individuals**

Three steps were required to identify and assign vocalizations to individuals. In the first, recordings of the first few minutes of echolocation clicks of photoidentified singletons were used to define the echolocation click IPI for that individual. We used *Rainbow Click* software [6–9] to identify and export waveforms of echolocation click trains. The IPIs of the clicks were then calculated using custom-written MATLAB routines which require approval of each automated click measurement by an observer (SG analyzed all recordings to remove observer differences). As the pulsed structure of the received click varies with the relative aspect of the whale to the hydrophone [10], previous studies [1,11–14] have used recordings made of the clicks during the first few minutes after a single animal dives (range: 2-18 minutes). This places the hydrophone such that it is “on-axis” and therefore at an aspect favourable to the accurate reception of the pulse structure [10]. Any click deemed to be “off-axis” based on visual inspection, and those for which there was a clear non-biological transient, such as wave and engine noise or hydrophone knocking, were omitted from further analyses. After approval, the routines calculated three estimates of the IPI using the autocorrelation function of the click waveform: 1) the time-delay of the function maximum, 2) the median time-delay among those of the five largest function values, and 3) the time-delay of the peak closest to the midpoint between the peaks which are at least 30% of the height of the function maximum. We then calculated a single mode over all three measures for all the clicks analyzed in a recording and if greater than 50% of the clicks were within 0.05msec of that mode, then we took that mode to be the measured IPI for that individual for that recording. The best-estimate echolocation click IPI for each whale in each year was calculated by taking the mode of the IPIs across its recordings over different days within the year. Echolocation click IPIs were calculated for each individual within each of the three units for which this was possible for each year from 2005-2010 (as inter-annual growth could have changed the values for an individual). The result of this initial step was a library of echolocation click IPIs which had been attributed to identified individuals for a given year.

Coda recordings were then analyzed using *Rainbow Click* software to determine the inter-click intervals (ICI, the time between the onset of one click and the onset of the next click) defining the temporal structure of all codas recorded. The codas were marked manually by a trained observer (SG analyzed all recordings) and the timing of the clicks within codas calculated by the software. Each coda could then be represented by the set of ICIs, using the absolute length of the intervals given previous research suggested that more information may be encoded in the absolute than when standardizing relative to total coda length [15]. Rare long coda types (>10 clicks; <5% of all codas recorded) were excluded from the analysis.

We then analyzed the coda recordings to determine the coda click IPI for each coda using the same *Matlab* routines as for the singleton echolocation clicks. Each coda was assigned an IPI by taking the mode over all three measures (as above) for all clicks within that coda. Just as when analyzing the IPI of echolocation clicks, only audible codas which had a clear structure were used such that codas that were off-axis or in which there was a clear non-biological transient were omitted from analyses. Finally, equipped with the library of echolocation click IPIs, we assigned a coda to an individual whale when its modal echolocation click IPI (derived from the recordings taken when the individuals were alone) was within 0.05 msec of the modal coda click IPI of a whale which was present at the time of recording and at least 0.1 msec different from the modal coda click IPI of every other whale present at the time of the recording. The details of how these cut-offs were determined are given in Schulz *et al.* [1]. Clusters were small [Mean = 1.75 individuals, SD=1.24, 16], and generally hundreds to thousands of meters apart. Thus only members of the photoidentified cluster were within a few hundred meters of the hydrophone and therefore recorded, given that codas are only audible through hydrophones that are near the surface over a few hundred meters (SG unpublished).

**Rhythm Plot for All Coda Types:**


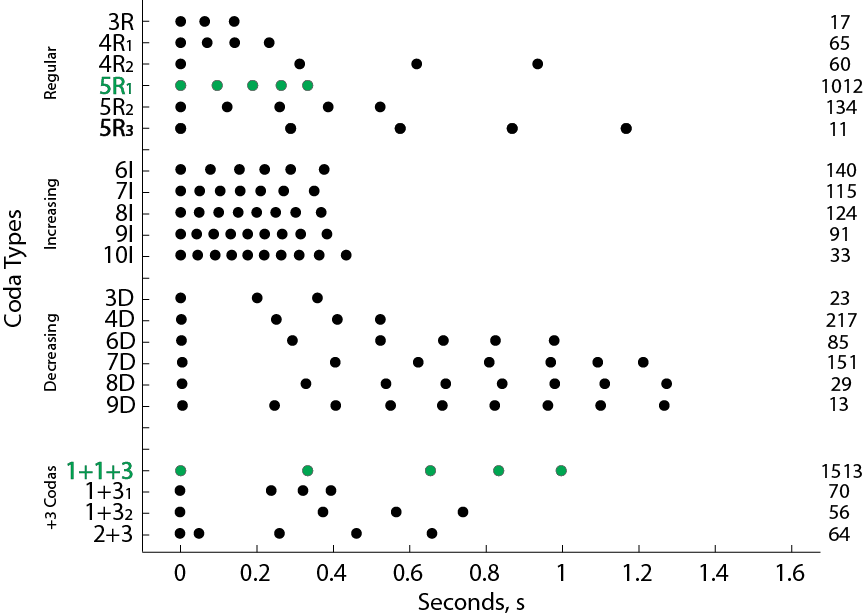


Figure S2 – Rhythm plot illustrating the mean timing of the clicks in all 21 coda types. Sample sizes on right axis and green fill denotes predominant coda types across units.

**Individual and Unit Level Coda Repertoire Discovery Curves:**

Discovery curves (Fig S2) suggest that repertoires at both the unit and individual level are well characterized. These curves suggest that having a sample of over 75 or 250 codas, for individuals and units respectively, provides a description of all but the very rare coda types. Antunes et al.’s [15] preliminary work indicated that the 5R codas varied among individuals, while the 1+1+3 did not, with a very small subset of data from one unit, and this larger dataset across multiple units confirms these findings. This suggests that our conclusions are likely robust to under sampling.


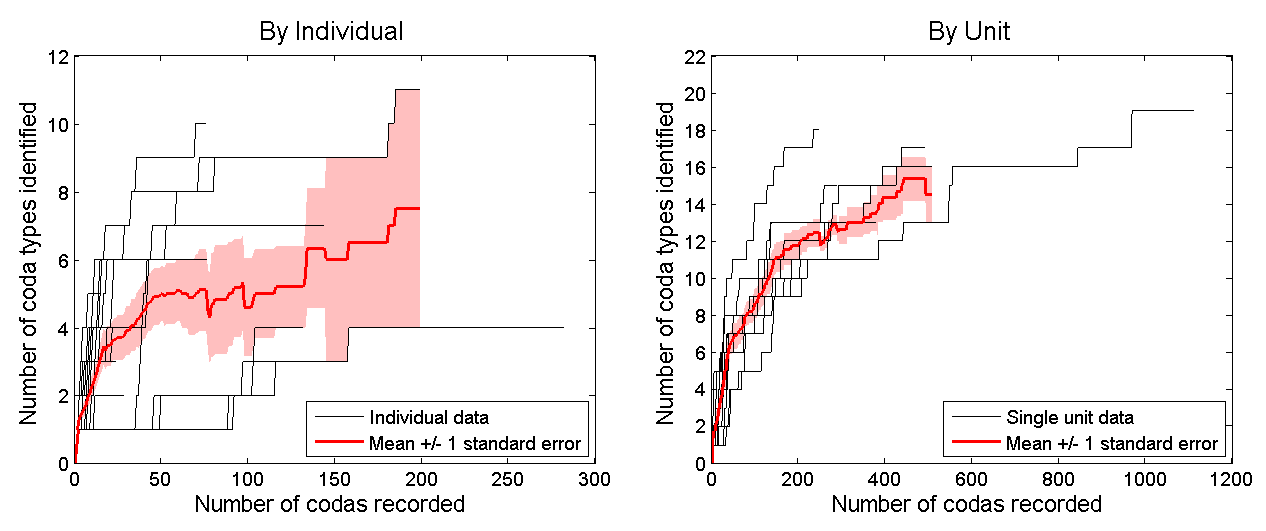


Figure S3 – Discovery curves of coda types by individual and unit.

**Similarity of Repertoires:**

*Methodological details of permutation test:*

Matrix correlations and Mantel tests with 10000 permutations [17,18] were used to test repertoire variation between similarity matrices of individuals within units, and between units, where a unit’s repertoire was simply all the codas produced by individuals belonging to that unit. We tested whether pair-wise similarities were higher between two days’ recordings of the same unit (same unit, different day – SUDD) compared with two days’ recordings of different units (different unit, different days – DUDD). Each day’s recordings were treated as independent in an attempt to account for any autocorrelation in coda production within a recording day. To do so, we tested the matrix of pair-wise similarities of each day’s recordings against a 0/1 matrix with 1 coding for SUDD and 0 coding for DUDD. If units, produced distinct repertoires, then the expectation is a significantly positive correlation between these matrices. We then took a similar approach for comparing individuals. In this case, we compared recordings of the same individual on different days with recordings of different individuals on different days.

*Similarities between years and across changes in social role:*

The mean similarities within and between years of an individual’s repertoire (Table S2) did not significantly differ (one-tail paired t-test by individuals with a null of no difference between means and an alternative that within year similarity would be greater: df = 11, p = 0.910 for the multivariate; p = 0.907 for the categorical), suggest that individuals do not change their vocal repertoire between years, at least over the six year duration of this study (2005-2010). Individual repertoires appear robust to changes in social role. Below, we provide examples of repertoires of three different females who changed social role during the study, either by giving birth and becoming a mother (Tables S4 and S5), or by losing a calf (Table S3).

**Table S2: Mean repertoire similarity of individuals within and between years.**

Multivariate similarity using absolute ICIs, Euclidean distances, and a basal similarity of 0.001. Categorical similarity using OPTICS classification.

| **Full Repertoire** | |  | **Multivariate** | | **Categorical** | |
| --- | --- | --- | --- | --- | --- | --- |
| **Unit** | **Individual** | **Years** | **Within**  **Year** | **Between**  **Years** | **Within**  **Year** | **Between**  **Years** |
| **F** | **Unit Means**  **SE** |  | 0.006  0.0006 | 0.006  0.0005 | 0.189  0.013 | 0.193  0.010 |
|  | **Pinchy** | 4 | 0.008 | 0.020 | 0.200 | 0.313 |
|  | **Mysterio** | 3 | 0.010 | 0.009 | 0.815 | 0.590 |
|  | **Fingers** | 4 | 0.018 | 0.025 | 0.475 | 0.499 |
|  | **Scar** | 3 | 0.006 | 0.005 | 0.338 | 0.373 |
| **J** | **Unit Means**  **SE** |  | 0.008  0.0011 | 0.009  0.0007 | 0.593  0.045 | 0.564  0.028 |
|  | **Jocasta** | 3 | 0.012 | 0.018 | 0.217 | 0.428 |
|  | **Oedipus** | 4 | 0.014 | 0.016 | 0.981 | 0.981 |
|  | **Sophocles** | 2 | 0.006 | 0.011 | 0.274 | 0.305 |
|  | **Laius** | 4 | 0.009 | 0.009 | 0.606 | 0.602 |
| **U** | **Unit Means**  **SE** |  | 0.013  0.0022 | 0.011  0.0016 | 0.254  0.038 | 0.206  0.034 |
|  | **Fork** | 2 | 0.025 | 0.025 | 0.321 | 0.455 |
|  | **Knife** | 2 | 0.054 | 0.052 | 0.711 | 0.737 |
|  | **Spoon** | 2 | 0.006 | 0.005 | 0.166 | 0.243 |
|  | **Canopener** | 2 | 0.020 | 0.016 | 0.405 | 0.497 |

Table S3 – Coda Repertoires of Fingers (Female #5722) from Unit F across years and after loss of calf in 2006. Note that in 2010, Fingers was recorded socializing with a mature male and sperm whales produce more codas when socializing [19].

| **Coda Type** | **2005**  **Mother** | **2008**  **No Role** | **2009**  **No Role** | **2010**  **No Role** | **Total**  **by Type** |
| --- | --- | --- | --- | --- | --- |
| **4D** | 44 | 25 | 16 | 84 | **169** |
| **1+1+3** | 0 | 2 | 0 | 4 | **6** |
| **7D** | 0 | 0 | 0 | 98 | **98** |
| **8D** | 0 | 0 | 0 | 8 | **8** |
| **Total By Year** | **44** | **27** | **16** | **194** | **281** |
| **Total Recording Days** | 5 | 2 | 1 | 4 |  |

Table S4 - Coda Repertoires of Jocasta (Female #5987) from Unit J across years and after birth of calf in 2009.

| **Coda Type** | **2007**  **No Calf** | **2008**  **No Calf** | **2010**  **Mother** | **Total**  **by Type** |
| --- | --- | --- | --- | --- |
| **4R1** | 0 | 0 | 1 | **1** |
| **5R1** | 6 | 12 | 15 | **33** |
| **5R2** | 0 | 0 | 5 | **5** |
| **1+1+3** | 0 | 8 | 14 | **22** |
| **6D** | 0 | 1 | 0 | **1** |
| **8I** | 0 | 1 | 1 | **2** |
| **Total By Year** | **6** | **22** | **36** | **64** |
| **Total Recording Days** | **1** | **2** | **3** |  |

Table S5 - Coda Repertoires of Pinchy (Female #5560) from Unit F across years and after birth of calf in late 2006.

| **Coda Type** | **2005**  **No Calf** | **2008**  **Mother** | **2009**  **Mother** | **2010**  **Mother** | **Total**  **by Type** |
| --- | --- | --- | --- | --- | --- |
| **5R1** | 29 | 10 | 6 | 10 | **55** |
| **5R2** | 15 | 0 | 0 | 0 | **15** |
| **1+1+3** | 43 | 2 | 0 | 7 | **52** |
| **7I** | 6 | 0 | 0 | 0 | **6** |
| **8I** | 7 | 0 | 0 | 2 | **9** |
| **9I** | 4 | 0 | 0 | 0 | **4** |
| **10I** | 2 | 0 | 0 | 0 | **2** |
| **Total By Year** | **106** | **12** | **6** | **19** | **143** |
| **Total Recording Days** | 6 | 2 | 2 | 3 |  |

*Similarity of Repertoires using alternative method:*

We used two measures to quantify codas and two different distance metrics to determine similarity between codas in multivariate space. The conclusions of the study are robust to the differing methods. In the main text, we present the Euclidean distance metric with Absolute ICI. Below, we present the results using the alternative metric, infinity-norm distance, and when ICIs are standardized by length [which conserves rhythm but discards tempo of codas as in 20]. For a complete description of the two methods see Rendell and Whitehead [3]. Mantel tests using the infinity-norm distance metric on standardized ICIs also confirmed that recordings of the same unit on different days are more similar than recordings of different units on different days (Table S3). Furthermore, Individuals still do not show differences in repertoire across years (Table S4).

Table S6- Mean repertoire similarities within and between units and individuals within units. Mantel test across all units (top row) has a null hypothesis that repertoire similarity between recordings of the same unit on different days (within unit) is the same as that between recordings of different units on different days (between units). Mantel tests between individuals within the three units (bottom 3 rows) have a null hypothesis that repertoire similarity between recordings of the same individual on different days (within individual) is the same as that between recordings of different individuals on different days (between individuals).

Multivariate similarity using absolute ICIs, Inifinity-norm, and a basal similarity of 0.001.

|  |  | **Multivariate Similarity** | | | |
| --- | --- | --- | --- | --- | --- |
| **Units** | **Repertoire** | **Within** | **Between** | **Matrix**  **Correlation** | ***P*** |
| **All** | **Full** | 0.013 | 0.010 | 0.13 | <0.001 |
| **F** | **Full** | 0.028 | 0.008 | 0.48 | <0.001 |
| **J** | **Full** | 0.028 | 0.022 | 0.13 | 0.03 |
| **U** | **Full** | 0.024 | 0.011 | 0.56 | <0.001 |

**Table S7 - Mean full repertoire similarity of individuals within and between years.**

Multivariate similarity using Standardized ICIs, infinity-norm distances, and b=0.001.

| **Full Repertoire** | |  | **Infinity Norm** | |
| --- | --- | --- | --- | --- |
| **Unit** | **Individual** | **Years** | **Within**  **Year** | **Between**  **Years** |
| **F** | **Unit Means**  **SE** |  | 0.010  0.0007 | 0.009  0.0005 |
|  | **Pinchy** | 4 | 0.013 | 0.021 |
|  | **Mysterio** | 3 | 0.026 | 0.017 |
|  | **Fingers** | 4 | 0.025 | 0.028 |
|  | **Scar** | 3 | 0.018 | 0.014 |
| **J** | **Unit Means**  **SE** |  | 0.023  0.002 | 0.023  0.001 |
|  | **Jocasta** | 3 | 0.018 | 0.019 |
|  | **Oedipus** | 4 | 0.029 | 0.034 |
|  | **Sophocles** | 2 | 0.022 | 0.016 |
|  | **Laius** | 4 | 0.027 | 0.026 |
| **U** | **Unit Means**  **SE** |  | 0.012  0.001 | 0.011  0.001 |
|  | **Fork** | 2 | 0.022 | 0.021 |
|  | **Knife** | 2 | 0.034 | 0.034 |
|  | **Spoon** | 2 | 0.008 | 0.008 |
|  | **Canopener** | 2 | 0.015 | 0.012 |

**Average-linkage hierarchical clustering dendrograms of repertoire similarity among individuals in two additional units:**

We used similarity matrices between repertoires to construct average-linkage clustering dendrograms and we tested their robustness using 1000 bootstrap replicates. At each bootstrap iteration, the codas from each repertoire were randomly sampled with replacement prior to calculating the pairwise repertoire similarities. For each branch, we counted the proportion of replicates in which the branch was reproduced. The cophenetic correlation coefficient (CCC) was also calculated to indicate how well the dendrogram represented the data. A CCC of over 0.8 is considered a “good” representation of the associations [21].

We were able to construct repertoires at the level of the individual for three units (F, J, and U). Unit F, with the largest sample size, was presented in the main text. Below are the coda type repertoires and similarity dendrograms for the two additional units.


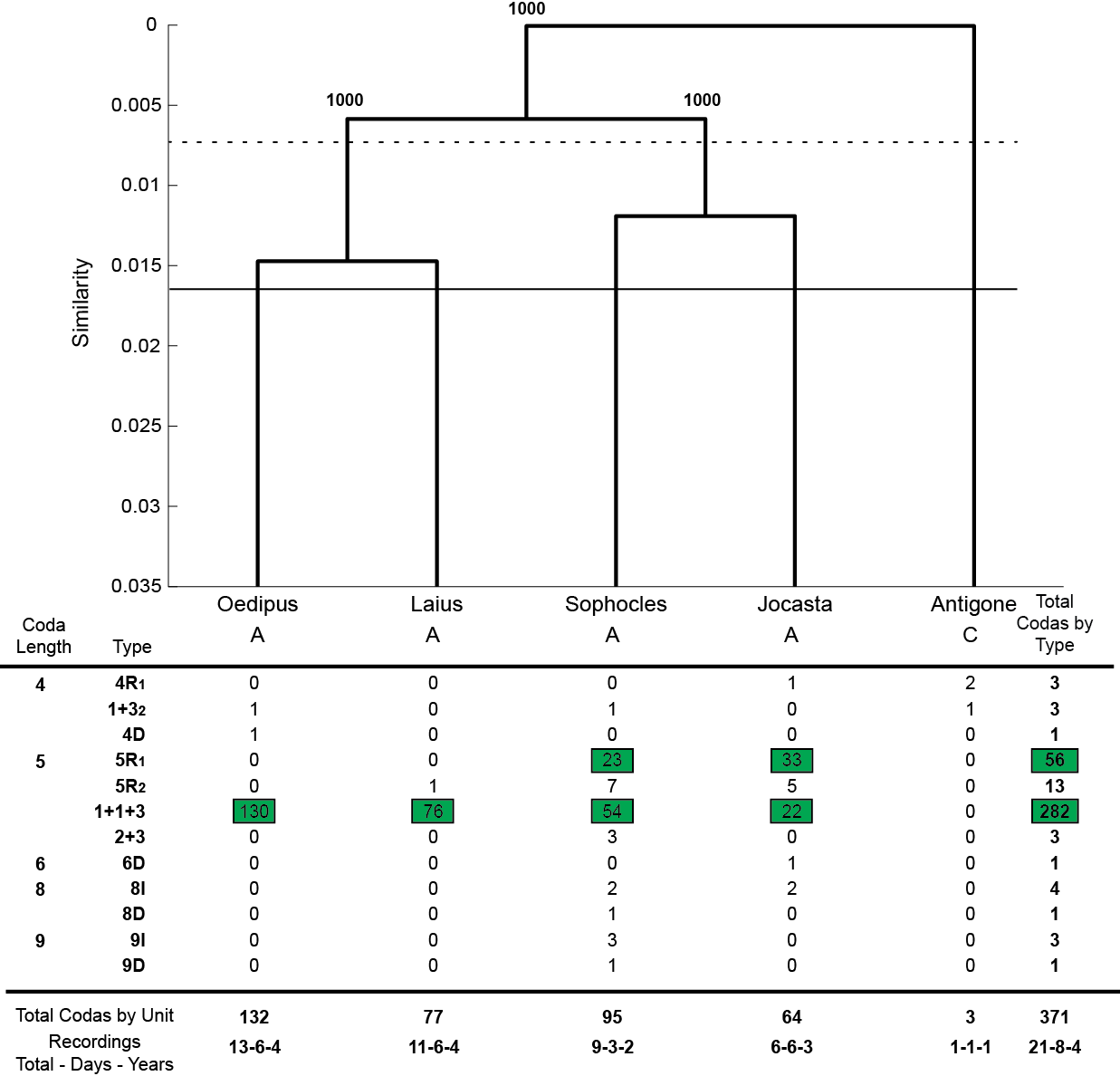


Figure S4: Coda repertoires of individuals in Unit J using Euclidean distances and absolute inter-click intervals with a basal similarity of 0.001 (top) and OPTICS classification into types (bottom). A cophenetic correlation coefficient of 0.9933 suggests this is a good representation. Letters denote age class (A – Adult, C – Calf). Horizontal rules indicate the mean between (dotted) and within (solid) unit similarities. All notations are as in Figure 1.


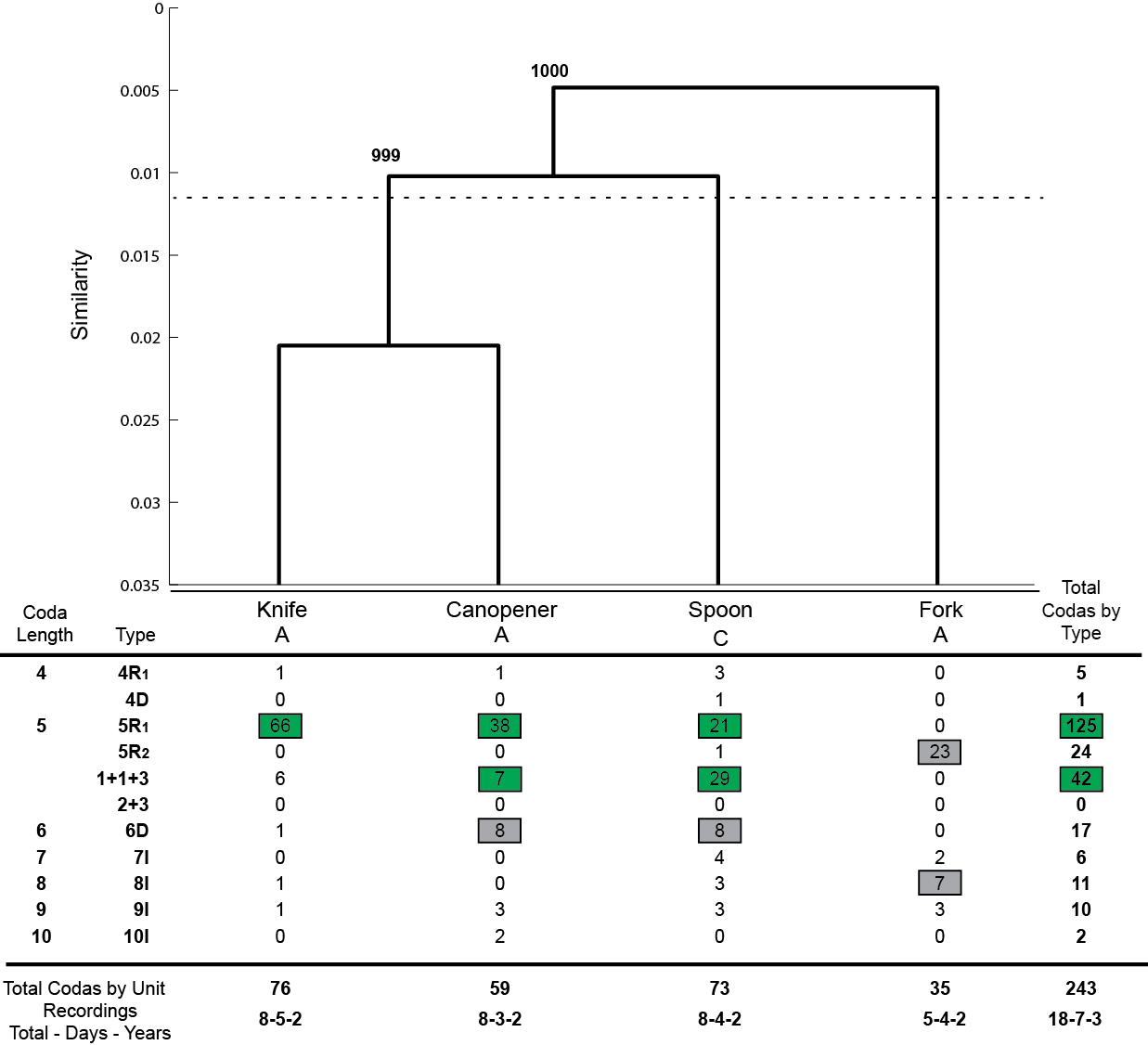


**Figure S5: Coda repertoires of individuals in Unit U using Euclidean distances and absolute inter-click intervals with a basal similarity of 0.001 (top) and OPTICS classification into types (bottom). A cophenetic correlation coefficient of 0.9924 suggests this dendrogram is a good representation of the differences in the repertoires. Letters denote age class (A – Adult, C – Calf). Horizontal rules indicate the mean between (solid) and within (dotted) unit similarities. All notations are as in Figure 1.**

Discriminant Function Analysis:

*Detailed Methodology:*

To determine whether particular coda types could be used to discriminate between units or individuals we undertook several discriminant function analyses on subsets of the data: only codas with 4 clicks, only codas with 5 clicks, only the ‘1+1+3’ codas (which is the most common coda type recorded in the study), all the ‘5R’ patterned codas [which Antunes et al. 15 found varied between individuals], and on the two most common types defined by OPTICS which have 5 clicks and a regular rhythm (‘5R1’ and ‘5R2’; we chose to exclude ‘5R3’ due to the small sample size and it being largely produced by only one unit). Linear discriminant function analysis was conducted on raw ICI data, with all codas classified as “noise” by OPTICS removed.

For clarity of comparison, discriminant coefficients were standardized by within-group estimates of variance [22]. We tested the statistical significance of the discriminant functions using the commonly used Wilks’ Lambda [which estimates the proportion of the variance in the dependent variables unaccounted for by the groupings, i.e. low values indicate large between-group differences, 23] with its approximated F-statistics and *p*-values. The DFA, error rates, and significance testing were calculated in R [ver. 3.0.1, 24] using the package MASS [ver. 7.3-27, 25].

Classification error rates were determined by leave-one-out cross-validation, in which one coda in the dataset is omitted at a time and the remaining codas are used to derive the discriminant functions which are then used to classify the omitted coda. Individual or unit level discrimination error rates were defined as the proportion of codas from a given unit or individual that were misclassified. Given that correct classification may be biased due to imbalances in the sample sizes between the groups being discriminated (i.e. the expectation under random assignment for *n* groups is not simply 1/*n* if the sample sizes are not equal), we further tested the classification by comparing the observed correct classification rate against those from 1000 randomized datasets. To do this we randomly re-assigned codas to either individual or unit, depending on the analysis level, while keeping the number of codas made by each individual or unit constant. We then recorded the mean correct classification from the 1000 random datasets.

*Supplemental Results:*

The results of the linear discriminant function analysis for all restricted datasets including those with no discrimination. The results of the 4-click codas and 5-click codas are in Table S6. Table S7 contains the results for the DFA on the 5R1 codas within units and table S8 provides the results for the analyses conducted on the 1+1+3 coda type.

Table S8 - Significance tests, DFA correct classification on observed data, and correct classification based on randomized data; where n is the number of units.

| **Coda Dataset** | **Wilks**  **Ʌ** | **Approx. F** | | **df** | ***p*** | | **Randomized Correct** | **DFA Correct Classification** | |  |
| --- | --- | --- | --- | --- | --- | --- | --- | --- | --- | --- |
| **4 Click Coda Types**  n=9 units | 0.045 | 106.42 | 24 | | | <0.001 | 54% | | 88% | |
| **5 Click Coda Types**  n=9 | 0.701 | 31.72 | | 32 | <0.001 | | 21% | 29% | |  |
| **1+1+3 Codas Only**  n=9 | 0.807 | 10.384 | | 32 | <0.001 | | 28% | 34% | |  |
| **5R1 Codas Only**  n=9 | 0.672 | 13.121 | | 32 | <0.001 | | 22% | 30% | |  |

Table S9 - Significance tests, DFA correct classification on observed data, and correct classification based on randomized data; where n is the number of individuals.

| **Coda Dataset** | **Wilks**  **Ʌ** | **Approx. F** | **df** | ***p*** | **Randomized Correct** | **DFA Correct Classification** |
| --- | --- | --- | --- | --- | --- | --- |
| **5 Click Coda Types**  n=19 | 0.370 | 15.83 | 72 | <0.001 | 15% | 29% |
| **1+1+3 Codas**  n=14 | 0.495 | 9.347 | 52 | <0.001 | 19% | 33% |
| **1+1+3 within Unit F**  n=6 | 0.507 | 11.22 | 20 | <0.001 | 37% | 57% |
| **1+1+3 within Unit J**  n=4 | 0.733 | 7.54 | 12 | <0.001 | 38% | 51% |
| **1+1+3 within Unit U**  n=3 | 0.827 | 0.920 | 8 | 0.505 | 63% | 58% |
| **5R1 & 5R2 Types**  n=14 | 0.172 | 15.311 | 52 | <0.001 | 18% | 46% |
| **All 5R1**  n=12 | 0.223 | 11.59 | 44 | <0.001 | 21% | 46% |
| **5R1 within Unit F**  n=3 | 0.526 | 6.156 | 8 | <0.001 | 73% | 83% |
| **5R1 within Unit J**  n=2 | 0.648 | 6.925 | 4 | <0.001 | 54% | 73% |
| **5R1 within Unit U**  n=3 | 0.373 | 19.138 | 8 | <0.001 | 51% | 78% |
| **5R1 within Unit V**  n=3 | 0.140 | 11.731 | 8 | <0.001 | 38% | 79% |

Table S10: Explained variance and standardized coefficients of discriminant functions. Bolded coefficients are the highest absolute coefficient for each discriminant function. Inter-click intervals (ICI) are numbered from first to last in the coda.

| **4 Click Codas**  **Discriminating Units** | Discriminant Functions | | | |  | |
| --- | --- | --- | --- | --- | --- | --- |
|  | 1 | 2 | 3 | |  | |
| Cumulative Variance Explained | 77.3 | 97.8 | 100 | |  | |
| Standardized Coefficients |  |  |  | |  | |
| ICI1 | **0.8060** | 1.6417 | 0.4067 | |  | |
| ICI2 | -0.7546 | **-2.0063** | **0.9488** | |  | |
| ICI3 | -0.7385 | 1.0698 | -0.7075 | |  | |
| **5 Click Codas**  **Discriminating Units** | Discriminant Functions | | | | | |
|  | 1 | 2 | 3 | | 4 | |
| Cumulative Variance Explained | 71.9 | 86.5 | 95.7 | | 100 | |
| Standardized Coefficients |  |  |  | |  | |
| ICI1 | -0.3121 | -1.0688 | -1.2638 | | 2.883 | |
| ICI2 | -0.0649 | 0.2350 | -0.2186 | | **-4.0398** | |
| ICI3 | **-0.4021** | **2.3186** | -0.2992 | | 0.9593 | |
| ICI4 | -0.2823 | -1.5627 | **1.7549** | | 0.2802 | |
| **All 5 Click Codas**  **Discriminating Individuals** | Discriminant Functions | | | | | |
|  | 1 | 2 | 3 | | 4 | |
| Cumulative Variance Explained | 61.5 | 83.9 | 93.4 | | 100 | |
| Standardized Coefficients |  |  |  | |  | |
| ICI1 | -0.2664 | 2.5459 | **3.7679** | | -0.0777 | |
| ICI2 | -0.3855 | **-3.8839** | -2.8340 | | 1.5062 | |
| ICI3 | **1.0129** | -0.4365 | 0.5887 | | **-1.9536** | |
| ICI4 | 0.5636 | 1.6661 | -1.2262 | | 0.9189 | |
| **5R1 Type Codas**  **Discriminating Individuals** | Discriminant Functions | | | | | |
|  | 1 | 2 | 3 | | 4 | |
| Cumulative Variance Explained | 80.5 | 92.5 | 98 | | 100 | |
| Standardized Coefficients |  |  |  | |  | |
| ICI1 | 0.2017 | **-0.9370** | -0.2867 | | -0.1255 | |
| ICI2 | -0.5912 | -0.1968 | 0.3929 | | -0.6964 | |
| ICI3 | -0.3756 | -0.1994 | 0.4824 | | **0.8598** | |
| ICI4 | **0.8427** | 0.0027 | **0.5363** | | -0.3501 | |
| **5R1&2 Type Codas**  **Discriminating Individuals** | Discriminant Functions | | |  | |  |
|  | 1 | 2 | 3 | | 4 | |
| Cumulative Variance Explained | 56.1 | 89.4 | 97.6 | | 100 | |
| Standardized Coefficients |  |  |  | |  | |
| ICI1 | 0.1617 | -0.2140 | **-1.3089** | | 0.3176 | |
| ICI2 | -1.0543 | 0.1279 | 0.2158 | | 1.1244 | |
| ICI3 | -1.0659 | 0.6881 | -0.1034 | | **-2.0798** | |
| ICI4 | **1.3410** | **-1.5112** | 0.8614 | | 0.6557 | |

Table S11: Explained variance and standardized coefficients of discriminant functions for individuals within units for the 5R1 coda. Bolded coefficients are the highest absolute coefficient for each discriminant function. Inter-click intervals (ICI) are numbered from first to last in the coda.

| **5R1 Type Codas**  **Individuals in Unit F** | Discriminant Functions | | |  | |  |
| --- | --- | --- | --- | --- | --- | --- |
|  | 1 | 2 |  | |  | |
| Cumulative Variance Explained | 86.7 | 100 |  | |  | |
| Standardized Coefficients |  |  |  | |  | |
| ICI1 | 0.5988 | 0.0562 |  | |  | |
| ICI2 | 0.3249 | 0.3964 |  | |  | |
| ICI3 | **0.6170** | 0.2378 |  | |  | |
| ICI4 | -0.1412 | **1.0098** |  | |  | |
| **5R1 Type Codas**  **Individuals in Unit J** | Discriminant Function | | |  | |  |
|  | 1 |  |  | |  | |
| Cumulative Variance Explained | 100 |  |  | |  | |
| Standardized Coefficients |  |  |  | |  | |
| ICI1 | -0.0063 |  |  | |  | |
| ICI2 | **0.7304** |  |  | |  | |
| ICI3 | 0.4314 |  |  | |  | |
| ICI4 | -0.4714 |  |  | |  | |
| **5R1 Type Codas**  **Individuals in Unit U** | Discriminant Function | | |  | |  |
|  | 1 | 2 |  | |  | |
| Cumulative Variance Explained | 92.7 | 100 |  | |  | |
| Standardized Coefficients |  |  |  | |  | |
| ICI1 | -0.3550 | 0.5345 |  | |  | |
| ICI2 | 0.5898 | -0.1069 |  | |  | |
| ICI3 | 0.4991 | -0.4950 |  | |  | |
| ICI4 | **-0.8512** | **-0.5790** |  | |  | |
| **5R1 Type Codas**  **Individuals in Unit V** | Discriminant Function | | |  | |  |
|  | 1 | 2 |  | |  | |
| Cumulative Variance Explained | 83.7 | 100 |  | |  | |
| Standardized Coefficients |  |  |  | |  | |
| ICI1 | **0.9431** | 0.0297 |  | |  | |
| ICI2 | 0.1143 | **-1.0081** |  | |  | |
| ICI3 | 0.1593 | -0.1198 |  | |  | |
| ICI4 | 0.8668 | 0.1031 |  | |  | |

Table S12: Explained variance and standardized coefficients of discriminant functions for individuals within units for the 5R1 coda. Bolded coefficients are the highest absolute coefficient for each discriminant function. Inter-click intervals (ICI) are numbered from first to last in the coda.

| **1+1+3 Type Codas**  **Discriminating Units** | Discriminant Function | | | | |
| --- | --- | --- | --- | --- | --- |
|  | 1 | 2 | | 3 | 4 |
| Cumulative Variance Explained | 63.3 | 25.0 | | 8.0 | 3.7 |
| Standardized Coefficients |  |  | |  |  |
| ICI1 | **1.1140** | -0.305 | | **-2.3891** | 0.5571 |
| ICI2 | -0.8166 | 0.1837 | | 1.5216 | **-2.0812** |
| ICI3 | -0.8930 | **0.9327** | | -0.3142 | 0.4975 |
| ICI4 | 1.0078 | 0.2457 | | 1.0791 | 0.7912 |
| **1+1+3 Type Codas Discriminating Individuals** | Discriminant Function | | | | |
|  | 1 | 2 | | 3 | 4 |
| Cumulative Variance Explained | 49.0 | 83.6 | | 93.3 | 100 |
| Standardized Coefficients |  |  | |  |  |
| ICI1 | -0.5372 | **-1.7530** | | **2.3514** | 0.9606 |
| ICI2 | **1.9620** | 1.6153 | | -1.1895 | **-1.6174** |
| ICI3 | 0.1029 | 0.5986 | | 0.0356 | 1.3495 |
| ICI4 | -0.7944 | -0.9960 | | -1.4346 | -0.1439 |
| **1+1+3 Type Codas**  **Individuals in Unit F** | Discriminant Function | | | | |
|  | 1 | 2 | | 3 | 4 |
| Cumulative Variance Explained | 77.8 | 96.6 | | 99.2 | 100 |
| Standardized Coefficients |  |  | |  |  |
| ICI1 | 0.6714 | **3.0840** | | **-**0.1333 | -1.1349 |
| ICI2 | **-2.3252** | -2.0434 | | 0.0748 | **1.7839** |
| ICI3 | -0.1139 | -0.1066 | | 1.1212 | -1.0272 |
| ICI4 | 1.1641 | -0.9657 | | **-1.4232** | -0.4673 |
| **1+1+3 Type Codas**  **Individuals in Unit J** | Discriminant Function | | | |  |
|  | 1 | 2 | | 3 |  |
| Cumulative Variance Explained | 86.7 | 99.5 | | 100 |  |
| Standardized Coefficients |  |  | |  |  |
| ICI1 | **1.0327** | 2.2117 | | -1.8322 |  |
| ICI2 | -0.0634 | **-2.7108** | | **2.9751** |  |
| ICI3 | -0.5513 | 1.2814 | | 0.5690 |  |
| ICI4 | 0.4008 | -0.2733 | | -1.3680 |  |
| **1+1+3 Type Codas**  **Individuals in Unit U** | Discriminant Function | |  | |  |
|  | 1 | 2 | |  |  |
| Cumulative Variance Explained | 90.9 | 100 | |  |  |
| Standardized Coefficients |  |  | |  |  |
| ICI1 | **-1.3683** | -0.0548 | |  |  |
| ICI2 | 0.7548 | -0.1044 | |  |  |
| ICI3 | 0.5585 | **1.1985** | |  |  |
| ICI4 | -0.4207 | -0.3927 | |  |  |

Discriminating Individuals within Units:

We were able to discriminate individuals by their 5R1 codas in four different units (F, J, U, and V). Below we present a plot of the discriminant function scores for the first two discriminant functions, a boxplot of the discriminant function which best splits the individuals, and a scatterplot of the 5R1 codas against all 5R1 codas analyzed with the axis selected as the two inter-click intervals with the greatest discriminatory power based on the linear discriminant analysis.

Unit F:

Figure S6 - LEFT: Discriminant function scores for all 5R1 Codas from two adult females and one juvenile male in Unit F, excluding the calves (83% correct, 73% when randomized, p=0.001). RIGHT: Boxplot of the first discriminant function for the two remaining individuals when female #5563 is excluded due to a small sample size (Correct 88%, 80% when randomized, p<0.0001)


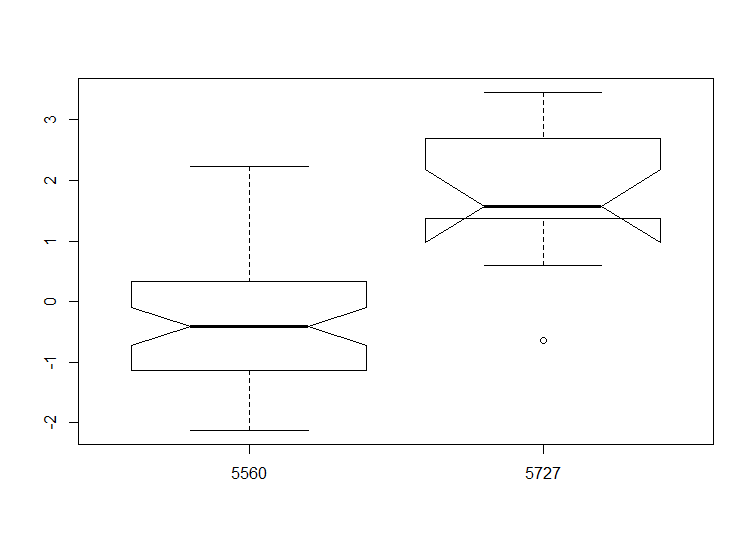

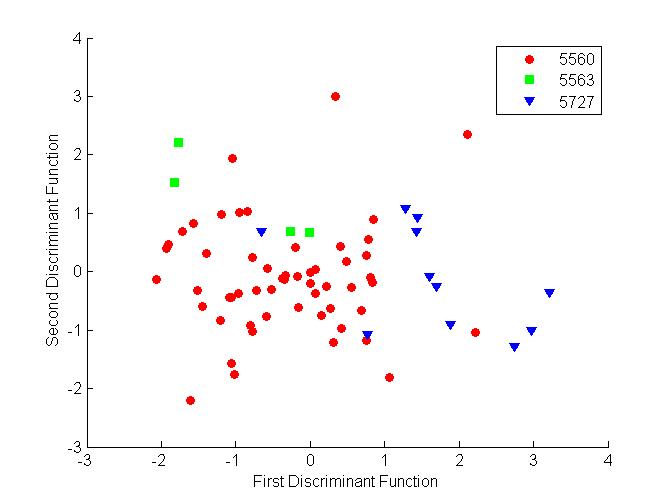


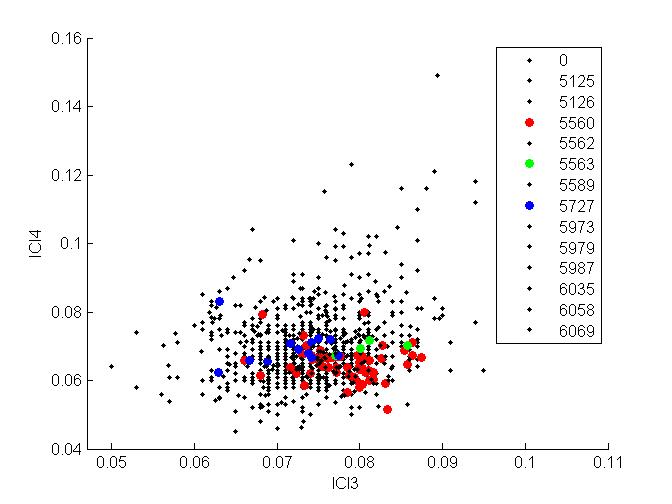


**Figure S7 - Scatterplot of All 5R1 codas produced by two adult females and one juvenile male in Unit F on all 5R1 sampled. ICI axes selected based on LDA coefficients.**


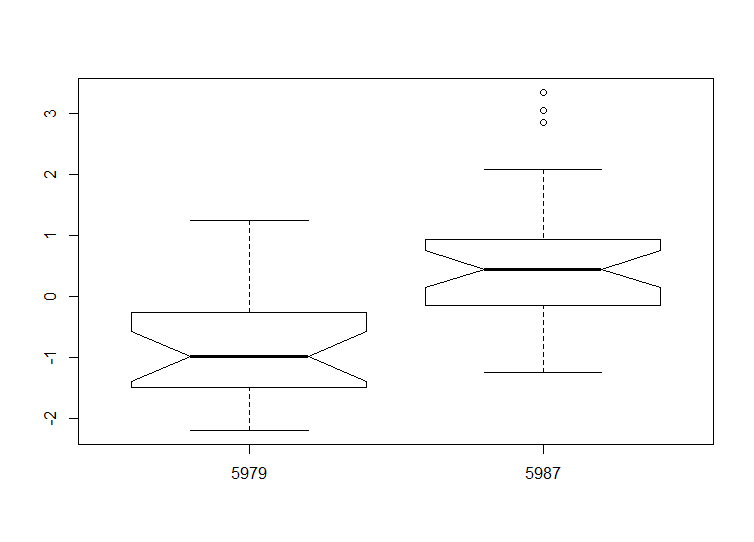
Unit J:

Figure S8 - Boxplot of first discriminant function scores for the two adult females in Unit J (73% correct, 54% when random, p=0.001)


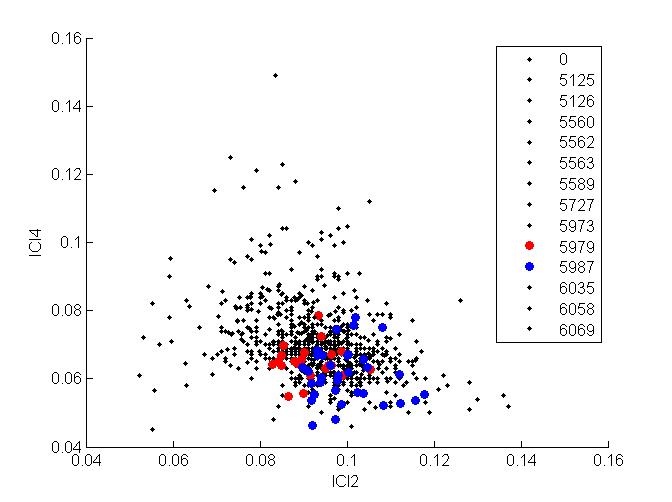


Figure S9 - Scatterplot of All 5R1 codas produced by two adult females in Unit J on all 5R1 sampled. ICI axes selected based on LDA coefficients.

Unit U:


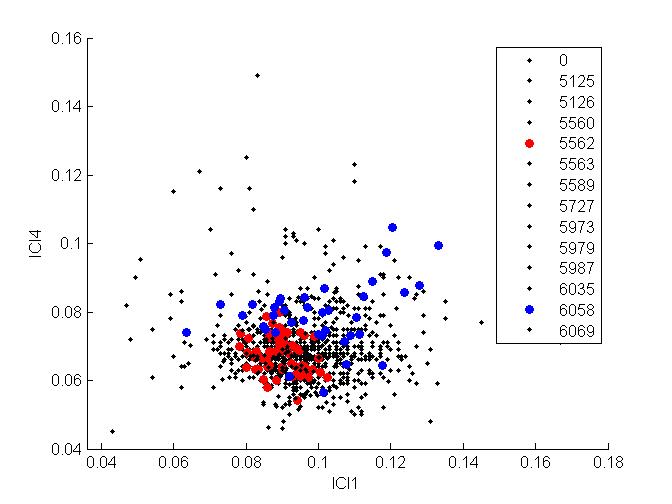


First Discriminant Function

Second Discriminant Function

Figure S10 - LEFT: Discriminant function scores for all 5R1 Codas from two adult females and one calf from Unit U (78% correct, 51% when randomized, p<0.0001). RIGHT: Boxplot of the first discriminant function for the two remaining adult females when the calf (#6035) is excluded (Correct 95%, 62% when randomized and p<0.0001).


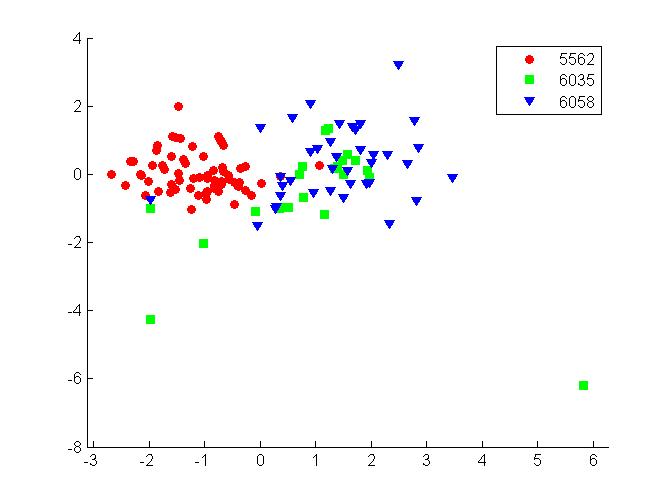

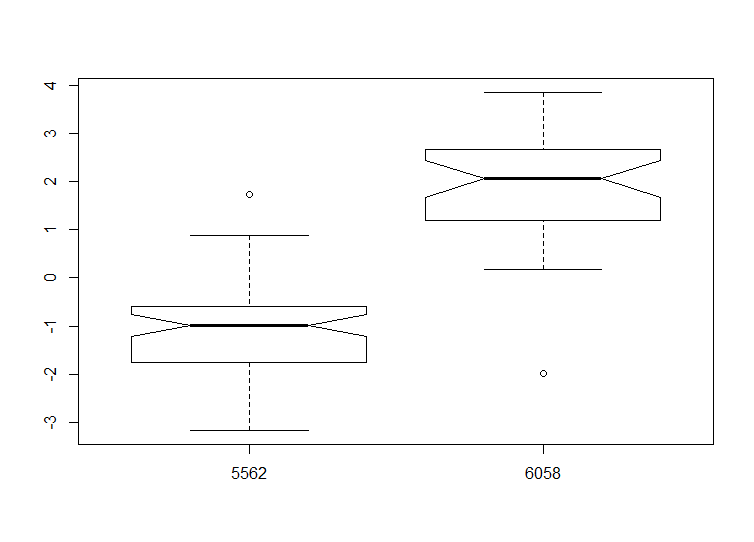


**Figure S11 - Scatterplot of All 5R1 codas produced by two adults in Unit U, excluding the calf, on all 5R1 sampled. ICI axes selected based on LDA coefficients.**


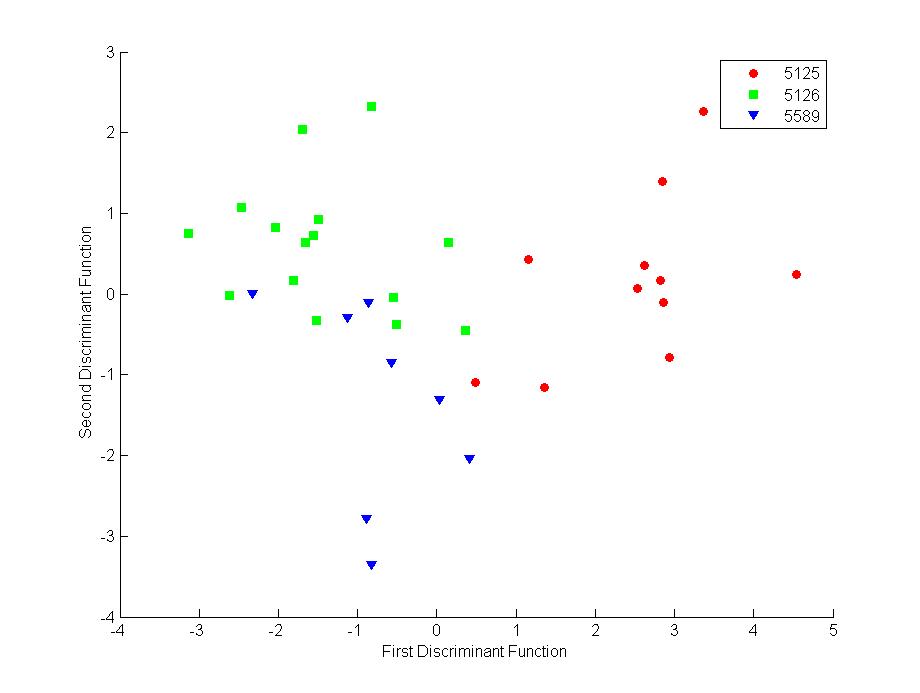
Unit V:

Figure S12 - Discriminant function scores for all 5R1 codas from three adult females in Unit V (79% correct, 38% when randomized, p=0.001).


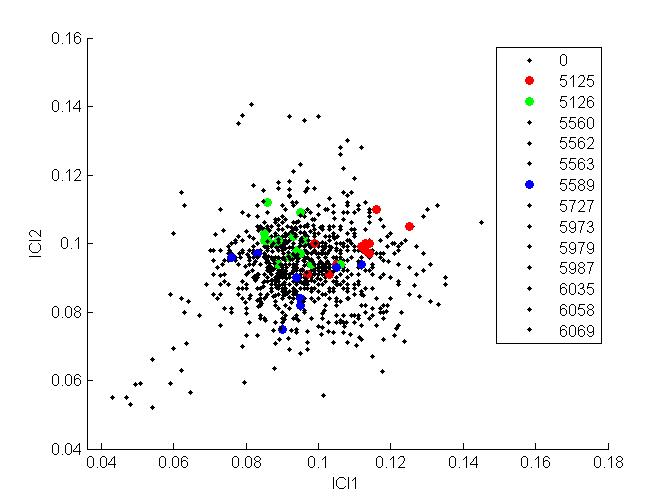


**Figure S13 - Scatterplot of All 5R1 codas produced by the three females from Unit V on all 5R1 sampled. ICI axes selected based on LDA coefficients. NOTE: in this case the two discriminant functions (above) line up well with ICI1 and ICI2 (this plot).**

References:

1. Schulz, T. M., Whitehead, H., Gero, S. & Rendell, L. 2011 Individual vocal production in a sperm whale (*Physeter macrocephalus*) social unit. *Mar. Mammal Sci.* **27**, 149–166.

2. Rendell, L. & Whitehead, H. 2003 Vocal clans in sperm whales (*Physeter macrocephalus*). *Proc. R. Soc. London Ser. B-Biological Sci.* **270**, 225–231. (doi:10.1098/rspb.2002.2239)

3. Rendell, L. & Whitehead, H. 2003 Comparing repertoires of sperm whale codas: A multiple methods approach. *Bioacoustics-the Int. J. Anim. Sound Its Rec.* **14**, 61–81.

4. Nanni, M. & Pedreschi, D. 2006 Time-focused clustering of trajectories of moving objects. *J. Intell. Inf. Syst.* **27**, 267–289. (doi:10.1007/s10844-006-9953-7)

5. Gero, S. 2012 On the dynamics of social relationships and vocal communication between individuals and social units of sperm whales. **PhD thesis**.

6. Gillespie, D. 1997 An acoustic survey for sperm whales in the Southern Ocean sanctuary conducted from the R/V Aurora Australia. *Reports Int. Whal. Comm.* **47**, 897–908.

7. Leaper, R., Gillespie, D. & Papastavrou, V. 2000 Results of passive acoustic surveys for odontocetes in the Southern Ocean. *J. Cetacean Res. Manag.* **2**, 187–196.

8. Jaquet, N., Dawson, S. & Douglas, L. 2001 Vocal behaviour of male sperm whales: Why do they click? *J. Acoust. Soc. Am.* **109**, 2254–2259.

9. Rendell, L. & Whitehead, H. 2004 Do sperm whales share coda vocalizations? - Insights into coda usage from acoustic size measurement. *Anim. Behav.* **67**, 865–874.

10. Zimmer, W. M. X., Madsen, P. T., Teloni, V., Johnson, M. P. & Tyack, P. L. 2005 Off-axis effects on the multipulsed structure of sperm whale usual clicks with implications for sound production. *J. Acoust. Soc. Am.* **118**, 3337–3345.

11. Gordon, J. C. D. 1991 Evaluation of a method for determining the length of sperm whales (*Physeter macrocephalus*) from their vocalizations. *J. Zool.* **224**, 301–341.

12. Goold, J. C. 1996 Signal processing techniques for acoustic measurement of sperm whale body lengths. *J. Acoust. Soc. Am.* **100**, 3431–3441.

13. Teloni, V., Zimmer, W. M. X., Wahlberg, M. & Madsen, P. T. 2007 Consistent acoustic size estimation of sperm whales using clicks recorded from unknown aspects. *J. Cetacean Res. Manag.* **9**, 127–136.

14. Rhinelander, M. Q. & Dawson, S. M. 2004 Measuring sperm whales from their clicks: Stability of the interpulse intervals and validation that they indicate whale length. *J. Acoust. Soc. Am.* **115**, 1826–1831.

15. Antunes, R., Schulz, T., Gero, S., Whitehead, H., Gordon, J. C. D. & Rendell, L. 2011 Individually distinctive acoustic features in sperm whale codas. *Anim. Behav.* **81**, 723–730.

16. Gero, S. et al. 2014 Behavior and social structure of the sperm whales of Dominica, West Indies. *Mar. Mammal Sci.* **30**, 905–922. (doi:10.1111/mms.12086)

17. Mantel, N. 1967 The detection of disease clustering and generalized regression approach. *Cancer Res.* **27**, 209–220.

18. Schnell, G. D., Watt, D. J. & Douglas, M. E. 1985 Statistical comparison of proximity matricies: applications in animal behaviour. *Anim. Behav.* **33**, 239–253.

19. Whitehead, H. & Weilgart, L. 1991 Patterns of visually observable behavior and vocalizations in groups of female sperm whales. *Behaviour* **118**, 275–296.

20. Moore, K. E., Watkins, W. A. & Tyack, P. L. 1993 Pattern similarity in shared codas from sperm whales (*Physeter catodon*). *Mar. Mammal Sci.* **9**, 1–9.

21. Bridge, P. D. 1993 Classification. In *Biological Data Analysis* (ed J. C. Fry), pp. 219–242. Oxford: Oxford University Press.

22. Mueller, R. O. & Cozad, J. B. 1988 Standardized Discriminant Coefficients: Which Variance Estimate Is Appropriate? *J. Educ. Behav. Stat.* **13**, 313–318. (doi:10.3102/10769986013004313)

23. Wilks, S. S. 1932 Certain generalizations in the analysis of variance. *Biometrika* **24**, 471–494.

24. R Core Team 2013 R: A language and environemnt for statistical computing. *R Found. Stat. Comput. Vienna, Austria* , URL http://www.R–project.org/.

25. Venables, W. N. & Ripley, B. D. 2002 *Modern applied statistics with S. Fouth Editition*. New York: Springer.
